# Immune monitoring using the predictive power of immune profiles

**DOI:** 10.1186/2051-1426-1-7

**Published:** 2013-06-27

**Authors:** Michael P Gustafson, Yi Lin, Betsy LaPlant, Courtney J Liwski, Mary L Maas, Stacy C League, Philippe R Bauer, Roshini S Abraham, Matthew K Tollefson, Eugene D Kwon, Dennis A Gastineau, Allan B Dietz

**Affiliations:** 1 Human Cellular Therapy Laboratory, Division of Transfusion Medicine, Department of Laboratory Medicine and Pathology, Mayo Clinic, 200 First Street, Rochester, MN, USA; 2 Division of Hematology, Department of Medicine, Mayo Clinic, 200 First Street, Rochester, MN, USA; 3 Biomedical Statistics and Informatics, Mayo Clinic, 200 First Street, Rochester, MN, USA; 4 Cellular and Molecular Immunology, Department of Laboratory Medicine and Pathology, Mayo Clinic, 200 First Street, Rochester, MN, USA; 5 Pulmonary and Critical Care Medicine, Mayo Clinic, 200 First Street, Rochester, MN, USA; 6 Department of Urology, Mayo Clinic, 200 First Street, Rochester, MN, USA

**Keywords:** Immunity, CD14, Biomarker, Monocytes, Myeloid suppressor, Treg, CD4, Survival, Cancer, Human

## Abstract

**Background:**

We have developed a novel approach to categorize immunity in patients that uses a combination of whole blood flow cytometry and hierarchical clustering.

**Methods:**

Our approach was based on determining the number (cells/μl) of the major leukocyte subsets in unfractionated, whole blood using quantitative flow cytometry. These measurements were performed in 40 healthy volunteers and 120 patients with glioblastoma, renal cell carcinoma, non-Hodgkin lymphoma, ovarian cancer or acute lung injury. After normalization, we used unsupervised hierarchical clustering to sort individuals by similarity into discreet groups we call immune profiles.

**Results:**

Five immune profiles were identified. Four of the diseases tested had patients distributed across at least four of the profiles. Cancer patients found in immune profiles dominated by healthy volunteers showed improved survival (p < 0.01). Clustering objectively identified relationships between immune markers. We found a positive correlation between the number of granulocytes and immunosuppressive CD14^+^HLA-DR^lo/neg^ monocytes and no correlation between CD14^+^HLA-DR^lo/neg^ monocytes and Lin^-^CD33^+^HLA-DR^-^ myeloid derived suppressor cells. Clustering analysis identified a potential biomarker predictive of survival across cancer types consisting of the ratio of CD4^+^ T cells/μl to CD14^+^HLA-DR^lo/neg^ monocytes/μL of blood.

**Conclusions:**

Comprehensive multi-factorial immune analysis resulting in immune profiles were prognostic, uncovered relationships among immune markers and identified a potential biomarker for the prognosis of cancer. Immune profiles may be useful to streamline evaluation of immune modulating therapies and continue to identify immune based biomarkers.

## Background

This work arose from frustration in the lack of consistent correlates between changes in the immune status of a patient and clinical outcome in immunotherapy clinical trials. We noted on several occasions in our own work that the typical approach to describing immunity was not adequate. For example, describing regulatory T cells (Tregs) in terms of its relationship to a parent population (such as Tregs as a percent of CD4+ cells) or grandparent population (such as lymphocytes) did not take into account if the patient was lymphopenic. This approach also ignored relationships between disparate lineages of the immune system. Every leukocyte can, at some level, interact with virtually all other leukocytes. If immunity is fundamentally based on the likelihood of different leukocytes to interact, then the frequency of each within a volume of blood is critical to predicting the nature and duration of an immune response.

Consequently, more global or systemic approaches are needed in order to understand the connections and interplay between various immune cells in humans [1]. Indeed, significant efforts are underway to measure the immunological changes in complex diseases [2]. Systems approaches to human immunity have included gene expression arrays [3], cytokine arrays [4], immunohistochemistry [5], or multiple phenotype analysis [6,7]. In this study, flow cytometry of whole blood was used to determine the frequency/μl in blood of defined immune markers (i.e. the frequency of Lineage^-^HLA-DR^-^CD33^+^ myeloid derived suppressor cells). Determining the number of cells for any immune marker per unit volume has two distinct advantages: it allows direct quantification of immune markers (cells/μl), and eliminates processing steps such as density gradient separation that may effect the analysis. More importantly, using quantitative analyses allows one to determine the relationships between all members of the immune system. We considered the combination of all immune markers that we measured within a patient as the patients’ immune phenotype.

To identify patients’ immune phenotypes common within a population, we calculated the immune phenotypes within the peripheral blood of healthy volunteers, in patients with malignant disease, and in patients with acute lung injury. Acute lung injury was used because it is a known immune suppressive condition associated with poor outcome. Using hierarchical clustering, we identified individuals with common immune phenotypes. These common immune phenotypes we have termed immune profiles. We have identified profiles within specific diseases as well as immune profiles shared across malignancies. Patients with an immune phenotype found in an immune profile shared by healthy volunteers survived longer independent of the underlying disease. Identification of immune profiles allowed discovery of novel relationships between immune cells. We report here a novel methodology to comprehensively characterize human immunity.

## Methods

### Patients and healthy volunteers

Samples were collected under approval of the Mayo Clinic Institutional Review Board. Specific enrollment criteria and previous results from typing peripheral blood for some glioblastoma (GBM) patients [8], non-Hodgkin lymphoma (NHL) patients [9], and healthy volunteers have been previously reported [8,9] and were used for reanalysis in this study. Briefly, GBM patient samples were collected prior to surgery with or without concurrent steroids. Non-Hodgkin lymphoma (NHL) patients were newly diagnosed or recently relapsed patients off all chemotherapy for at least eight weeks. Patients with metastatic renal cell carcinoma (RCC) had newly diagnosed or recent relapsed disease and had samples taken before cytoreductive nephrectomy. Ovarian cancer patients (OVA) were newly diagnosed or relapsed with no chemotherapy for the prior 8 weeks. Cancer patient demographics can be found in Additional file 1: Table S1. Acute lung injury (ALI) patients who presented with at least one risk factor for acute lung injury/acute respiratory distress syndrome [10] within 12 hours of admission and/or recognition of the diagnosis were selected. Inclusion criteria are from the American-European Consensus Conference [11] and consists of acute onset of hypoxemia (PaO_2_/FiO_2_ ≤ 300, acute lung injury; ≤200, acute respiratory distress syndrome) and diffuse radiologic infiltrates in the absence of left atrial hypertension. Risk factors include pneumonia, sepsis, pancreatitis, shock, aspiration, high risk surgery, and high risk trauma [12]. The age of the healthy volunteers was not different from each group except for RCC (Additional file 2: Figure S1).

### Flow cytometry of whole blood

Peripheral blood was used as the source for antibody staining as previously described [8,13]. Immune markers identified included granulocytes, lymphocytes, monocytes (identified by forward and side scatter), CD3^+^ T cells, CD19^+^ B cells, (CD56^+^CD16^+^) NK cells, CD4^+^ T cells, regulatory T cells (CD4^+^CD25^+^CD127^lo^), CD86^+^ total monocytes and CD14^+^HLA-DR^lo/neg^ immunosuppressive monocytes. Antibody reagents are listed in Additional file 3: Table S2. BD TruCount™ tubes (BD Biosciences, San Jose, CA) were used to collect cell counts/μl of blood for T, B and NK cells. This 4-color assay test does not require the exclusion of dead cells. The remaining markers were measured as a percent of these cells by adding fluorochrome-conjugated antibodies directly to 50-100 μl of whole blood and incubated for 15-20 minutes at room temperature in the dark. Red blood cells were lysed with BD FacsLysis Solution per manufacturer’s instructions. Cells were centrifuged, washed with phosphate buffered saline, and fixed in 4% para-formaldehyde. Data was acquired on a BD FACSCalibur™ flow cytometer calibrated the day of use and analyzed with Cell Quest, Multiset (BD), and/or Flowjo (Ashland, OR) software. The cell counts of granulocytes, lymphocytes, and monocytes were combined in each profile and the average was plotted as pie graphs to represent the total population of circulating immune cells. The cell counts of CD4^+^, CD8^+^, B, NK, cells, regulatory T cells, and CD14^+^ HLA-DR^+^ and HLA-DR^lo^ monocytes were combined and the average plotted as a pie graph to represent the total circulating mononuclear cells. CD8 cells were reported as the difference of CD3 and CD4 cells. CD14^+^ HLA-DR^+^ and HLA-DR^lo/neg^ monocytes were calculated from the percentage of CD14^+^ monocytes of total monocytes cell count (by forward/side scatter) and multiplied by the percentage of HLA-DR^+^ and HLA-DR^lo/neg^ cells.

### Multiparameter analysis and hierarchical clustering

Immune marker values were either measured directly in cells/μl or converted into cells/μl using the T, B, and NK counts. Mean values of each immune marker were determined using the values from 40 healthy volunteers (HV). Each immune marker for each individual (HV and patients) was then normalized by dividing the individual value by the mean value of healthy volunteers of that marker. The marker ratios for each volunteer and patient were imported into Partek Genomics Suite 6.5 software (Partek Inc., St. Louis, MO) and log-transformed for hierarchical clustering. Hierarchical analysis was performed by unsupervised agglomerative Euclidean average linkage clustering. Principal component analysis was performed using the Scatter plot view in the Partek program. Immune phenotypes as defined in this paper were the number and composition of circulating white blood cells within an individual. An immune profile was a group of immune phenotypes (containing a minimum of seven members) with as few dendrogram branches as possible. Additionally, all diseased members within a profile were compared to diseased members of other profiles (unless indicated) to determine profile differences.

### Statistical analyses

Values for subgroups of data were tested for statistical significance using the two-tailed non-parametric Mann-Whitney test for unpaired samples, the non-parametric Spearman correlation test for correlative analyses, and the Fisher’s 2 × 2 or 3 × 3 exact test for distribution between profiles. Cox models were used to identify prognostic factors for overall survival, where the models were adjusted for age and stratified by disease. The method of Contal and O’Quigley was used to determine a best cut-point for the CD4^+^/CD14^+^HLA-DR^lo/neg^ monocyte ratio [14]. Overall survival was evaluated using standard Kaplan-Meier methods. All statistical analyses and graphs were performed using Prism, version 5.0 software (GraphPad Software, San Diego, CA) and SAS software (SAS Institute Inc., Cary, NC).

## Results

### Identification of distinct immune profiles within diseases

We assessed the number and relative composition of ten immune markers in peripheral blood of HV and patients. These markers provide a comprehensive overview of the immune system with unambiguous gating strategies or have clearly defined functions related to immune suppression [8,15]. To cluster potentially similar immune phenotypes, cell counts were measured or calculated, normalized to that of healthy volunteers, and analyzed using hierarchical clustering and principal component analysis.

**Figure 1:**
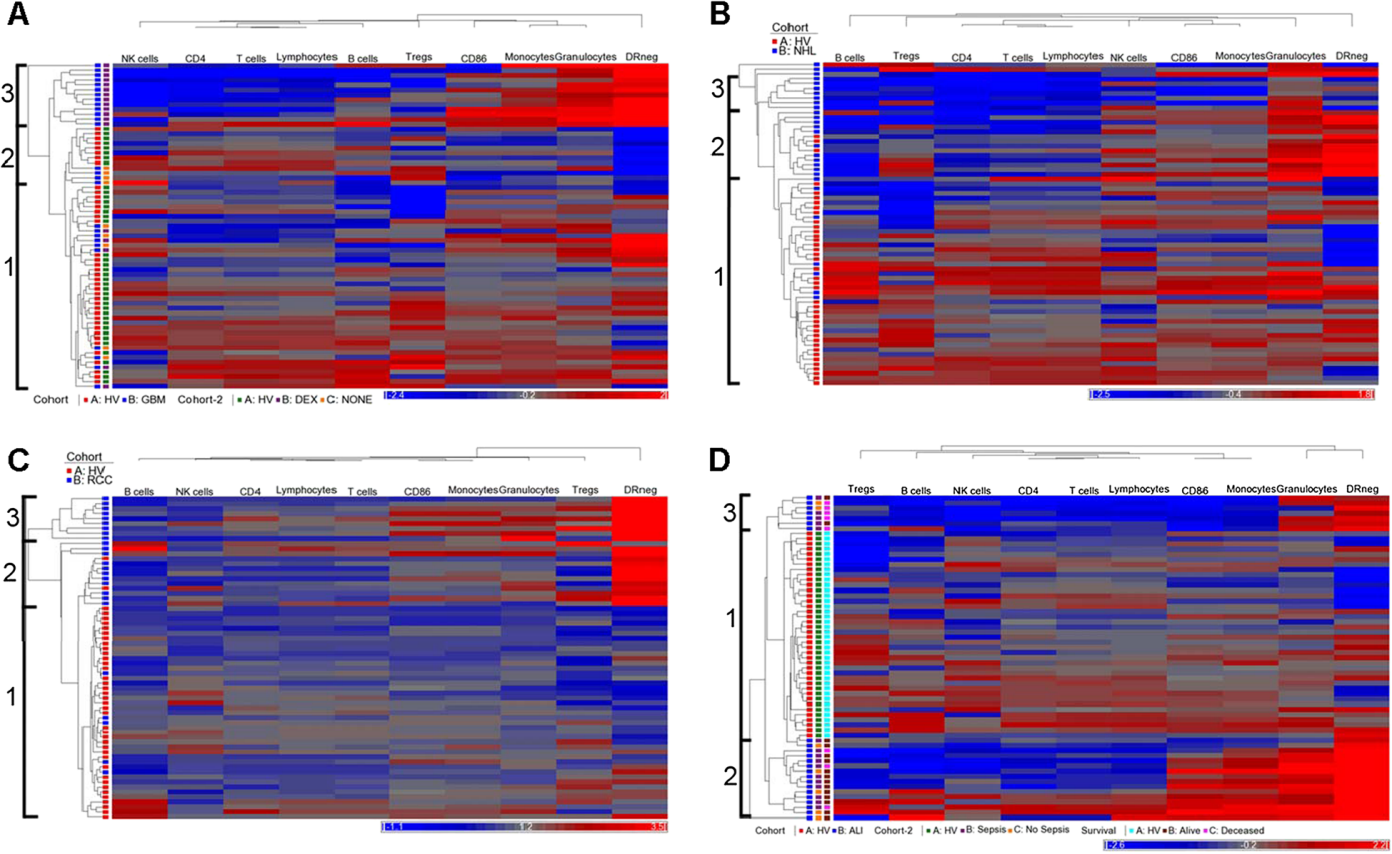
**Hierarchical clustering identifies immune profiles within patient groups.** Peripheral blood leukocyte populations were measured by flow cytometry. The number of cells/μl for each marker was determined directly or converted from TruCount tubes. All phenotype values were normalized against the mean of similarly measured and converted healthy volunteers (n = 40). Unsupervised clustering was performed using ten immune markers for each disease group (blue) and healthy volunteers (red; HV). The same HV cohort was used for all clustering analysis. Identification of major clusters is indicated at left. A row represents one subject and a column represents one of ten markers measured. The horizontal bar below each plot indicates immune markers decreased (blue) or increased (red) over the mean of the healthy volunteer cohort. **(A)** Clustering of patients with glioblastoma (GBM; n = 27). GBM patients were further identified based on the presences of pre-operative dexamethasone (DEX; purple), its absence (NONE; orange). HV indicated in green. **(B)** Clustering of patients with non-Hodgkin lymphoma (NHL; n = 28). **(C)** Clustering of renal cell carcinoma patients (RCC; n = 25). **(D)** Clustering of patients with acute lung injury with or at risk for sepsis (ALI; n = 23). ALI patients are further identified as those with (purple) or without (orange) confirmed sepsis as well as those that did (brown) or did not (pink) survive the episode.

We clustered immune phenotypes within individual malignancies using HV as a control group for clustering. We performed unsupervised hierarchical clustering on 27 GBM patients with 40 HV (Figure 1A). Three clusters of patients with similar immune systems (profiles) were identified. Profile 1 contained 32 HV, 5 dexamethasone (DEX) treated patients, and 5 untreated GBM patients; Profile 2 contained 8 HV and 4 untreated patients and Profile 3 contained only 13 DEX treated GBM patients (p = <0.0001; Fisher’s 3 × 3 exact test). The segregation of these patients based on DEX treatment agreed with our conventionally analyzed immune markers of these patients [8]. Thus, hierarchical clustering identified previously known informative subgroups.

We analyzed patients with NHL, RCC, and OVA in a similar manner with the same set of healthy volunteers. We assigned three profiles in NHL and RCC patients and in each case, the majority of patients clustered in profiles separate from those with the HV profile (Profile 1) (Figure 1B &1C). Alternatively, the OVA patients clustered across profiles with within the healthy volunteers, (Additional file 4: Figure S2).

We analyzed the immune phenotypes of ALI. Many critically ill ALI patients have an initially strong pro-inflammatory response but can quickly fall into a prolonged anti-inflammatory state called immune paralysis [16,17]. We felt that this patient population would test this approach in a non-malignant condition. There were three clearly identifiable profiles. Profile 1 contained all HV and 2 ALI patients, Profile 2 contained 7 ALI patients (5 septic), and Profile 3 contained 16 ALI patients (11 septic) (Figure 1D). Profiles 2 and 3 did not show differences in the distribution of septic patients. However, patients in Profile 2 exhibited a lower survival rate than profiles 1 and 3 in that 71% of patients in profile 2 died from their condition, where 19% of patients in Profiles 1 and 3 died (p = 0.026; Additional file 5: Figure S3). Together this data suggests that hierarchical clustering can identify unique immune profiles for each disease group, profiles that correlate with overall immune status, and clinical outcome.

### Identification of distinct immune profiles across several diseases

We noted that assigned profiles differed regarding the underlying immune characteristics. (i.e. Profile 2 in GBM does not share the same immune characteristics as Profile 2 in NHL or RCC). The power of hierarchical clustering to informatively segregate immune phenotypes is dependent on the number of the individuals used in the analysis. To identify immune profiles that represent a common immune status, we repeated these assays in an analysis that combined all healthy volunteers and patients.

**Figure 2:**
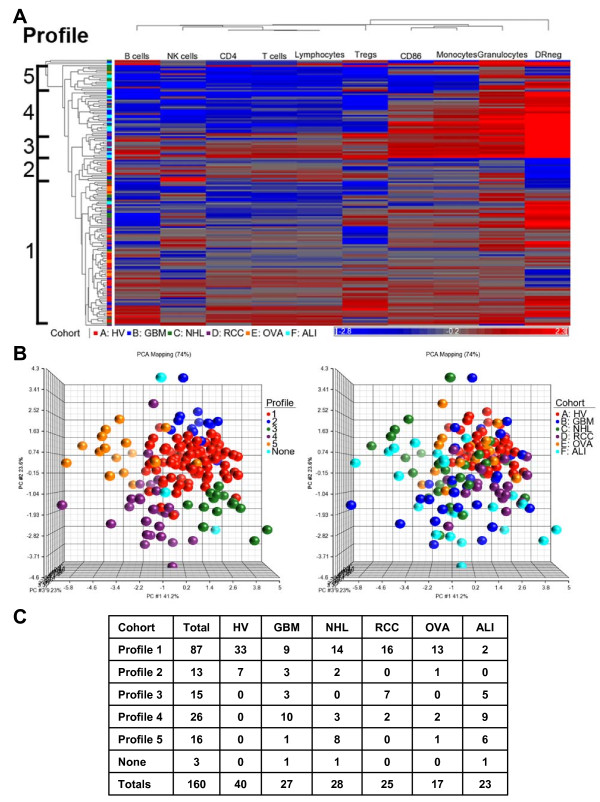
**Distinct immune profiles are shared across patient populations.** Ten immune markers for each individual from healthy volunteers (n = 40) and patients (n = 120) were used as sample data for combined clustering analysis **(A)** Hierarchical clustering dendrogram of patients and HV. Profiles were assigned based on the separation of the clustering trees. **(B)** Principal component analysis scatter view plots. Colors were based on clustering profile (left) and also disease group (right). None = no assigned profile (1 GBM, 1 NHL, and 1 ALI patient) **(C)** Distribution of patients and volunteers within each profile.

Five major profiles with at least 10 patients were identified (labeled 1-5) based on the distances of separation of the branches of the dendrogram (Figure 2A). Principal component analysis represented individuals by profile (Figure 2B, left scatter plot) and disease group (Figure 2B, right scatter plot). Disease distribution is shown in Figure 2C. All HV were clustered within two immune profiles. The distribution of subjects among immune profiles was different for each pathology. Thus, distinct profiles of immunity are shared across diseases with disease specific profile distribution.

**Figure 3:**
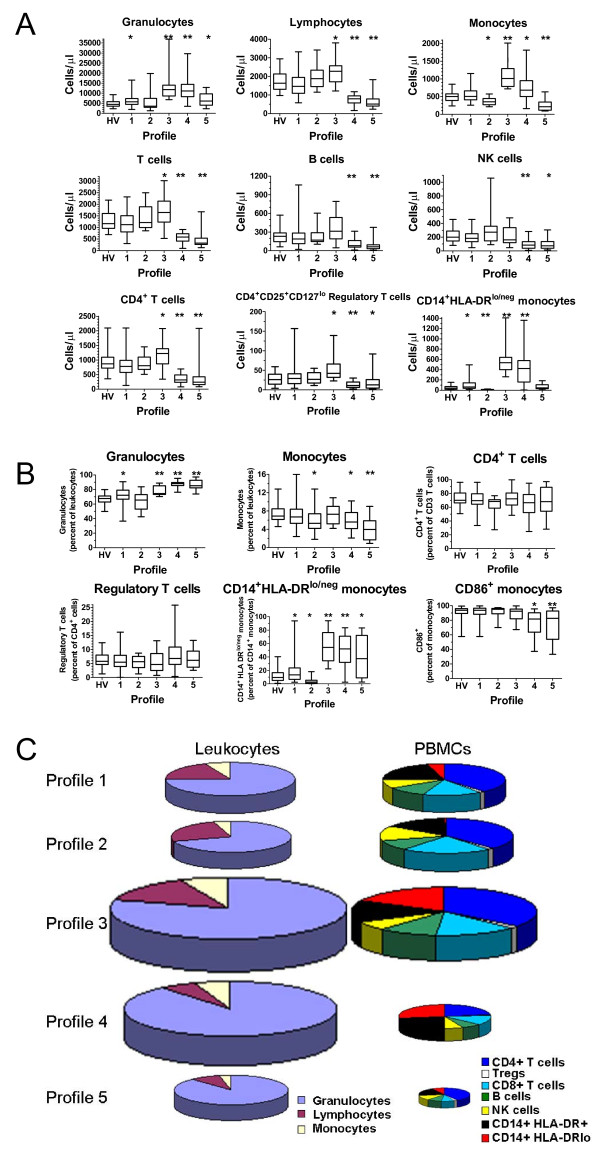
**Immune profiles are distinct in relative and absolute composition of immune markers.** Immune markers from each subject in a designated profile were evaluated for statistical significance. **(A)** Comparisons of immune marker cell counts. Box and whisker plots show mean, maximum, and minimum values for each data set. Box represents the 25^th^ to 75^th^ percentile range. HV = healthy volunteers only. * = p < 0.05 and ** = p < 0.0001. Each profile was compared to the healthy volunteer cohort. **(B)** Comparisons of immune marker percentages. Box and whisker plots show mean, maximum, and minimum values for each data set. Box represents the 25^th^ to 75^th^ percentile range. HV = healthy volunteers only. * = p < 0.05 and ** = p < 0.0001. Each profile was compared to the healthy volunteer cohort. **(C)** Visualization of immune profiles size and composition. To develop a picture of the composition within immune profiles, selected immune markers (in cells/μl) were totaled within an individual, and the mean of the individuals were calculated within a profile. The average profile was used to reconstruct the exemplar within each profile. Graph size represents total leukocytes/μl for the average profile relative to the average of Profile 1. Graphs on the left show the three major components of leukocytes. Graphs on the right show selected proportions of mononuclear cells. See Additional file 8: Figure S4 for graphical characterization and statistical analysis of this data.

To confirm the uniqueness of each immune profile, we compared the values of each marker to the values of the markers from other profiles or to only the pooled healthy volunteers (Figure 3A and Additional file 6: Table S3). The values of the markers from patients within profiles 1 and 2 (where HV typically segregate) were most similar to the HV pooled group. When compared to the pooled HV, Profile 1 had fewer lymphocytes and elevated CD14^+^HLA-DR^lo/neg^ monocytes; profile 2 had fewer monocytes and fewer CD14^+^HLA-DR^lo/neg^ monocytes when compared to the pooled HV profile. Profile 3 had elevated granulocytes, monocytes, and lymphocytes (mainly the T cell compartment), elevated regulatory T cells and CD14 + HLA-DR^lo/neg^ monocytes. Profile 4 had elevated granulocytes and monocytes but decreased lymphocytes (including T, B, and NK cells), decreased CD4+ T cells and elevated CD14 + HLA-DR^lo/neg^ monocytes. Abnormally low monocytes and lymphocytes including CD4+ T cells were present in patients in Profile 5. These data confirm that the profiles, as determined by hierarchical clustering, correspond to unique immune characteristics despite the disease of the patients. In addition, while we observed specific immune differences in each of the disease groups as outlined in Additional file 7: Table S4, when these profiles were analyzed together, common patterns of immune characteristics were identified independent of disease group.

Immune markers are most commonly reported as a ratio to a related population. We obtained the values from each immune phenotype as more typically reported and compared that data with values from the other profiles or the pooled healthy volunteers (Figure 3B). This analysis confirmed that the profiles were identifying subjects with similar immune statuses representing both absolute and relative differences.

Our data allowed the reconstruction of the average leukocyte composition of blood existing within a profile for the entire leukocyte compartment and peripheral blood mononuclear cells (PBMC; Figure 3C). The size of the pie chart reflects the relative quantity of cells per fixed unit of blood relative to Profile 1. For example, Profile 3 had almost twice as many total leukocytes as Profile 1 (p < 0.0001 and Additional file 8: Figure S4) and Profile 4 had over 1.5 times that of Profile 1 (p < 0.0001 and Additional file 8: Figure S4). The direction of the absolute change in total cells/μl was similar in all profiles except profile 4 where the leukocyte population increased while the PBMC population decreased. The magnitude of the difference also differed in the PBMC pools with Profile 3 having 1.5 times the amount of PBMCs than Profile 1 (p < 0.0001 and Additional file 8: Figure S4) while Profile 5 had less than half of PBMCs than Profile 1 (p < 0.0001 and Additional file 8: Figure S4). These results suggest that there exists peripheral blood immune profiles shared across disease states and that these profiles consist of changes in the absolute and relative quantities of individual white blood cells.

### Immune profiles correlate with patient outcome

**Figure 4:**
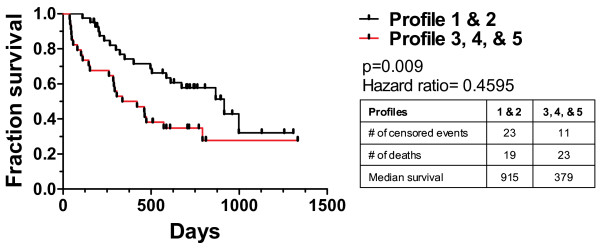
**Survival of cancer patients categorized by immune profiles.** Individual patients with GBM, NHL, or RCC with survival data were assigned a profile from Figure 2. Patients were pooled into profile groups independent of underlying disease. Profiles 1 and 2 were grouped as they represent the only profiles seen in healthy volunteers and compared to the survival of patients with profiles of 3, 4, and 5. P values were calculated by the Mantel-Cox log rank test while adjusting for the contributions of age and disease.

In previous studies, we have shown single immune markers with prognostic effects in single diseases [9,18]. Here, we have shown that immune profiles were predictive of survival in ALI (Additional file 5: Figure S3). We used those diseases with survival data (GBM, NHL, and RCC patients) to see if immune profile predicted survival across cancer diagnosis. We categorized patients by immune profile adjusting for age and cancer diagnosis. We grouped profiles most closely resembling normal immune system and compared them to those that do not (Figure 4). The median overall survival of patients in profile 1 and 2 (915 days, n = 42) was almost two and a half times as long as those in the other immune profiles (379 days; n = 34, p = 0.009). Similar analysis within disease groups did not identify survival associated profiles (Additional file 5: Figure S3). However, the studies suffered from insufficient sample size. The power of this approach will improve as the sample size increases. The approach presented here has the potential to segregate patients based solely on an unbiased immune status and identify those with the worst prognosis independent of underlying disease.

### Identification of related immune markers using hierarchical clustering

**Figure 5:**
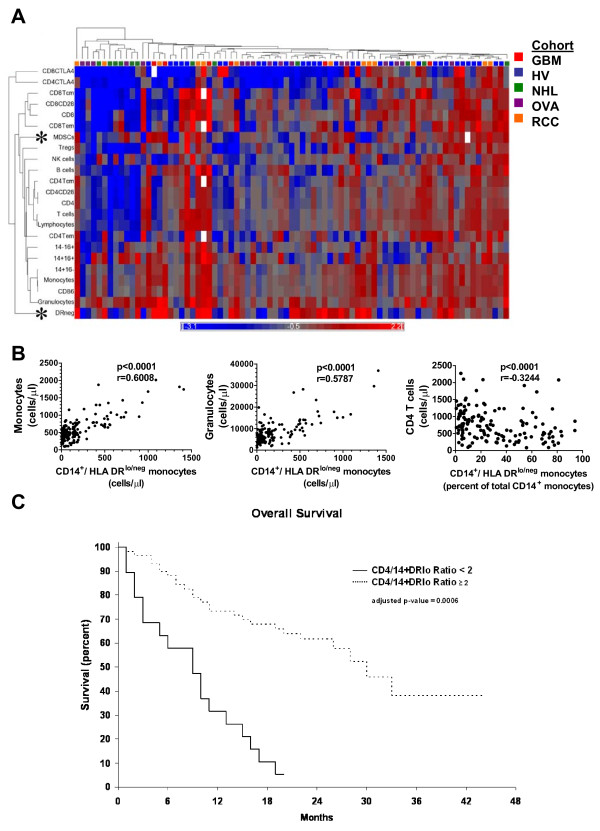
**Hierarchical clustering identifies relationships between immune markers. (A)** An additional 13 immune markers were added to the original ten. Cancer patients (n = 48) and healthy volunteers (n = 31) were analyzed as in Figure 1. White boxes in the dendrogram indicate that data was not collected or deemed suitable for analysis. For correlative studies, values from all 160 healthy volunteers and patients were used. **(B)** Monocytes and granulocytes were plotted against CD14^+^HLA-DR^lo/neg^ monocyte cell counts and CD4 T cell counts were plotted against the percentage of CD14^+^HLA-DR^lo/neg^ monocytes of total CD14^+^ monocytes. P values were calculated using the Spearman non-parametric correlation test. **(C)** The overall survival for GBM, NHL, and RCC patients was adjusted for age and disease. A ratio of cells/μl of CD4 T cell to CD14^+^HLA-DR^lo/neg^ monocytes was calculated for each patient and subgrouped into those above or below a cut-off value of 2.0 Patients with ratio at or above 2.0 (similar to healthy volunteers; dashed line) had a median overall survival of 30 months. and those below 2.0 (solid line) had a median overall survival of 9 months.

In addition to clustering individuals into immune profiles, hierarchical clustering identifies immune markers related by similar presence across immune profiles. A subset of our patients had been typed with 23 immune markers. We repeated our previous analysis to search for related expression. Some relationships observed were expected including the presence of CD4^+^ T cells segregating with CD28^+^CD4^+^ T cells or CD14^+^CD16^-^ monocytes with CD86^+^ monocytes (Figure 5A). However, some were novel such as granulocytes with CD14^+^HLA-DR^lo/neg^ or T_regs_ segregating independently from CD4^+^ cells. Lineage^-^HLA-DR^-^CD33^+^ myeloid derived suppressor cells (MDSCs) clustered independently of both granulocytes and monocytes, suggesting independent regulation. Thus, this analysis produced correlative evidence of similar or disparate regulation of certain leukocytes in humans.

A marker clustering largely by itself was the CD14^+^HLA-DR^lo/neg^ phenotype. Previous work identified CD14^+^HLA-DR^lo/neg^ monocytes as a predictor of poor prognosis and powerful mediators of immune suppression in GBM [8], NHL [9], chronic lymphocytic leukemia [18] melanoma [19], and renal cell cancer [20]. These cells are a subset of monocytes that express low or no HLA-DR (See Additional file 9: Figure S5 for a representative gating strategy). This phenotype had one of the largest degrees of change in both relative and absolute terms in our analysis (Figure S3 and Additional file 7: Table S4). To investigate if this hierarchical clustering could identify interesting immune marker correlations within the immune system we performed correlation analysis with two closely related marker and one marker segregating at a distant to the CD14^+^HLA-DR^lo/neg^ phenotype. CD14^+^HLA-DR^lo/neg^ monocyte cell counts positively correlated with total monocyte and granulocyte counts, markers that closely segregated to the CD14^+^HLA-DR^lo/neg^ (Figure 5B). We had previously found that CD4+ T cell counts were inversely correlated to the percentage of CD14^+^HLA-DR^lo/neg^ monocytes (of total CD14^+^ monocytes) in GBM patients. Here, in a larger cohort of subjects including both volunteers and cancer patients, CD4^+^ cells segregated distally from the CD14^+^HLA-DR^lo/neg^ phenotype and were inversely correlated to the percentage of CD14^+^HLA-DR^lo/neg^ monocytes (p < 0.001; Spearman r = -0.3244). The data suggest that CD14^+^HLA-DR^lo/neg^ monocytes are independently regulated are at odds with CD4+ T cells, and are important to the characterization of the overall status of the immune system. The analysis also identified key inverted relationships that might lead to improved understanding the relationships between leukocytes within the immune system.

### The CD4^+^/CD14^+^HLA-DR^lo/neg^ ratio is a prognostic biomarker in cancer patients

We chose the inverted relationship between CD14^+^HLA-DR^lo/neg^ and CD4^+^ cells identified above to determine if selected but disparate informative markers could describe immune status. Although lymphocytes counts have proven to be useful prognostic markers in some cancer populations [21,22] analysis of the individual markers have rarely identified a survival difference. We calculated the ratio of the number of CD4^+^ T cells to the number of CD14^+^HLA-DR^lo/neg^ monocytes (cells/μl). The 40 healthy volunteers had a mean CD4^+^/CD14^+^HLA-DR^lo/neg^ ratio of 39.8 (median 22.5) with a minimum of 3.9. The GBM, NHL, and RCC patients were subgrouped into those with high or low ratio, with a cut-point ratio of 2.0. We analyzed the overall survival of GBM, NHL, and RCC patients with high and low ratio using multivariate analysis to control for age and disease type. The median overall survival for patients with a ratio above 2.0 was 30 months (n = 68) compared to 9 months for patients with a low ratio (n = 39; p = 0.006 by multivariate analysis) (Figure 5C). This ratio is potentially a strong predictive biomarker for risk stratification and prognosis.

## Discussion

We have presented a novel approach to comprehensively describe the immune system based on whole blood flow cytometry of patients with a diverse pathology, determining the number of cells/μl for the major leukocyte components and hierarchical clustering of the generated data. The power of bioinformatics to cluster by similarity is related to the number of samples included in the analysis, the disease stratification and how consistent the individuals are within a profile. We acknowledge that the data presented here needs to be followed by expanded sampling of more disease states and healthy volunteers. However, the technical approach used (whole blood flow cytometry with cell quantitation), combined with a consensus antibody and gating strategy, could be used to establish a comprehensive analysis of peripheral blood immunity with thousands of patients and healthy volunteers to extract novel relationships between immunity and disease. The strength of our study results from 1) direct staining of fresh un-manipulated whole blood, 2) the use of an unbiased approach looking at multiple immune markers, 3) reporting cell populations as cell counts (cells/μl) to enumerate populations more accurately, and 4) a data set of healthy volunteer to determine the degree of change of immune markers. By combining these principles with standard gating strategies and patient health annotation, a large multi-institutional database could be established to provide a powerful resource for understanding human immunology.

Our description of different diseases sharing immune profiles has direct implications on the development and evaluation of immune modulating drugs. Patients likely have one of several distinct immune profiles influenced by their underlying disease, yet with a distinct response to immune modulating drugs correlating more to their immune profile than to the underlying disease. Therefore, it may be more informative to develop immune modulating drugs based on immune profiles rather than disease pathology.

The immune system can respond quickly to injury or infection, as well as having dynamic oscillations as part of homeostatic maintenance [23]. Our data provides a model of a stable immune system existing in a series of stable states analogous to the free energy landscapes described in other complex systems [24]. Free energy landscapes exist in complex multimodal systems where certain conditions produce nodes of stability. If this model were applied to the immune system, there may be stable immune states corresponding to key activation or response states such as acute or chronic infections or wound repair. Therefore, it’s possible that when cancer pushes the immune system to change, it changes to a finite number of pre-determined immune states. Evidence from gene expression analysis supports this concept in that the immune response to severe trauma in humans may be affected by distinct inflammatory states that lead to different outcomes [25]. Longitudinal sampling as well as characterization of other disease states (autoimmunity, wound repair, etc) will be required to fully test this concept. We don’t know if these states represent an active pathology within the disease or if they are biomarkers secondary to the pathology. Like any new method to identify biomarkers, this system will require testing to validate this approach.

Lastly, this work is limited to the immune markers used to describe the system. Further work is needed to characterize the markers that impact the clustering of the immune profiles. Likewise, addition of chemokines, cytokines or other global measures of immunity could add to this analysis. Like all clustering approaches, a few of the markers play a large role in identification of the profiles. The CD14^+^HLA-DR^lo/neg^ immune marker segregated independently and inversely from CD4+ T cells. The inverse relationship between these two cells types has not been explicitly described before and these results suggest that the CD4/CD14^+^HLA-DR^lo/neg^ monocyte ratio is an important biomarker for cancer prognosis. Further validation in future prospective clinical trials will be required. The advantages of viewing the whole immune system, the identification of shared immunity across diagnosis, and the potential for the discovery of novel relationships within the immune system should drive studies to describe the number and function of immune profiles.

## Conclusions

We have developed a method to comprehensively describe peripheral blood immunity using whole blood quantitative flow cytometery combined with bioinformatics. This approach provided a fresh perspective of immunity, allowing reconstruction of the absolute and relative distribution of leuckocytes. Organizing patients according to their underlying immune similarities (immune profiles) was prognostic independent of their underlying disease. Clustering identified relationships between luekcocytes populations supporting hypothesis development around these populations. For example, Lineage^-^HLA-DR^-^CD33^+^ myeloid derived suppressor cells (MDSCs) clustered independently of granulocytes, monocytes and CD14^+^HLA-DR^neg^ cells suggesting independent regulation. Clustering was used to identify a novel prognostic biomarker using CD14^+^HLA-DR^-^ and CD4^+^ cells. We believe that comprehensive immune profiling as described has the potential to rapidly accelerate the development of immune modulatory therapies.

## Abbreviations

GBM: Glioblastoma multiforma; NHL: Non-Hodgkin’s lymphoma; RCC: Renal cell carcinoma; Treg: Regulatory T cells; OVA: Ovarian cancer patients; ALI: Acute lung injury; T: T lymphocytes; B: B lymphocytes; NK: Natural killer cells; HV: Healthy volunteers; DEX: Dexamethasone; PBMC: Peripheral blood mononuclear cells.

## Competing interests

MP Gustafson, Y Lin, and AB Dietz have applied for a patent regarding this technology. No other authors have a conflict or competing interest in this manuscript.

## Authors’ contributions

MPG designed study, performed research, analyzed data and wrote manuscript, YL collected data, analyzed data and edited the paper, BLP performed statistical analysis, CJL collected the data, MLM, collected and audited the data, SCL provided critical insight into gating and whole blood flow expertise, PRB provided patient access and diagnosis, RSA provided critical flow cytometry expertise and edited the paper, MKT provided collected data and provided patient access, EDK provided patient access, edited the paper, DAG wrote and edited the paper, and ABD designed the research, and wrote and edited the paper. All authors read and approved the final manuscript.

## Supplementary Material

Additional file 1: Table S1 Patient demographics. Click here for file

Additional file 2: Figure S1 Age of healthy volunteers and patients. Box and whisker plots show the mean, 25^th^ and 75^th^ percentile, and the range of ages for each cohort. HV- healthy volunteers; GBM- glioblastoma multiforme; NHL-non Hodgkin’s lymphoma; RCC-renal cell carcinoma; OVA-ovarian cancer; ALI- acute lung injury. Asterisk indicates = p < 0.05 vs. HV. Click here for file

Additional file 3: Table S2 Antibodies and reagents used for flow cytometry. Click here for file

Additional file 4: Figure S2 Hierarchical clustering of ovarian cancer patients. OVA patients were subject to profiling analysis as in Figure 1. Identification of major clusters is indicated at left. A row represents one subject and a column represents one of ten markers measured. The horizontal bar below each plot indicates immune markers decreased (blue) or increased (red) over the mean of the healthy volunteer cohort. (n = 17 OVA and n = 40 HV). Click here for file

Additional file 5: Figure S3 Survival of patients categorized by immune profile. GBM, NHL, RCC, or ALI patients were categorized to a profile in Figure 1. For each disease, cohorts of patients sharing a profile were plotted for their survival. Note: only profiles with more than three individuals were plotted. Click here for file

Additional file 6: Table S3 P values of the differences in phenotype expression between each immune profile. Click here for file

Additional file 7: Table S4 Frequency of phenotypes for each pathology in this study. Click here for file

Additional file 8: Figure S4 Immune profile dependent differences in the number of leukocytes and mononuclear cells per μL of blood. Numerical representation of pie charts represented in Figure 3C. Box and whisker plots show the mean, 25^th^ and 75^th^ percentile, and the range of cell counts for each cohort. Differences (p < 0.0001) compared to profile 1 are indicated by ** above the profile. Click here for file

Additional file 9: Figure S5 Gating strategy for CD14^+^HLA-DR^lo/neg^ monocytes. After preparing the samples for CD14 and HLA-DR whole blood flow cytometry, a gate was placed on the intermediate side scatter and forward scatter cell population. A second gate on cells with low forward scatter and CD14+ was placed. A bivariate plot of CD14 vs. HLA-DR was created. The fraction of the cells in the HLA-DR^lo/neg^ is recorded. A representative plot from a normal healthy volunteer and patient are shown. Click here for file
