# Supplementary material for: Immune monitoring using the predictive power of immune profiles
Source: J Immunother Cancer. 2013 Jun 27;1:7. doi: 10.1186/2051-1426-1-7 (PMC4266565; doi:10.1186/2051-1426-1-7)
Supplement: Additional file 1: Table S1 — Patient demographics. [file 2051-1426-1-7-S1.docx]

Table S1. Patient demographics.

| Lymphoma | Total N=28 |  |
| --- | --- | --- |
|  | Age; average years (range) | 58.2 (20 – 81) |
|  | Gender; (%) |  |
|  | Female | 10 (35.7%) |
|  | Male | 18 (64.3%) |
|  | Histology; (%) |  |
|  | DLBCL | 13 (46.4%) |
|  | FL | 8 (28.6%) |
|  | LPL | 1 (3.6%) |
|  | MALT | 1 (3.6%) |
|  | Mantle | 4 (14.2%) |
|  | SLL | 1 (3.6%) |
|  | Stage; Ann Arbor (%) |  |
|  | I-II | 8 (40%) |
|  | III-IV | 20 (60%) |
|  | IPI (%) |  |
|  | 0-1 | 11 (39.3%) |
|  | 2 | 6 (21.4%) |
|  | 3 | 8 (28.6%) |
|  | 4-5 | 3 (10.7%) |
|  | Newly diagnosed; (%) | 4 (20%) |
|  | Relapsed disease; (%) | 24 (80%) |
|  | Number of prior treatments; median (range) | 2.5 (1-12) |
|  | Months from last treatment; median (range) | 10.5 (1-276) |
|  |  |  |
| Glioblastoma | Total N=27 |  |
|  | Age; average years (range) | 57.9 (25 - 81) |
|  | Gender (%) |  |
|  | Female | 8 (29.6%) |
|  | Male | 19 (70.4%) |
|  | Newly diagnosed; (%) | 20 (74.1%) |
|  | Relapsed disease (%) | 7 (25.9%) |
|  | Number of prior treatments; median (range) | 1.3 (1-2) |
|  | Months from diagnosis to recurrence; median (range) | 12.0 (4-26) |
|  |  |  |
| Renal Cell Carcinoma | Total N=25, all newly diagnosed, Stage IV |  |
|  | Age; mean (range) | 62.2 (38 -78) |
|  | Gender (%) |  |
|  | Female | 7 (28%) |
|  | Male | 18 (72%) |
|  | Histology (%) |  |
|  | Clear cell | 22 (88%) |
|  | Papillary | 3 (12%) |
|  |  |  |
| Ovarian Cancer | Total N=17, all newly diagnosed |  |
|  | Age; mean (range) | 56.1 (29-78) |
|  | Histology (%) |  |
|  | Serous | 11 (64.7%) |
|  | Mucinous | 2 (11.8%) |
|  | Clear cell | 1 (5.9%) |
|  | Mixed containing serous | 2 (11.8%) |
|  | Mixed others | 1 (5.9%) |
|  | Stage (%) |  |
|  | Stage I-II | 3 (%) |
|  | Stage III-IV | 14 (%) |

DLBCL, diffuse large B-cell lymphoma; FL, follicular lymphoma; LPL, lymphoplasmacytic lymphoma; MALT, mucosa associated lymphoid tissue lymphoma; SLL, small lymphocytic lymphoma; IPI, international prognostic index.
