# Supplementary figures and images for: Immune monitoring using the predictive power of immune profiles
Source: J Immunother Cancer. 2013 Jun 27;1:7. doi: 10.1186/2051-1426-1-7 (PMC4266565; doi:10.1186/2051-1426-1-7)

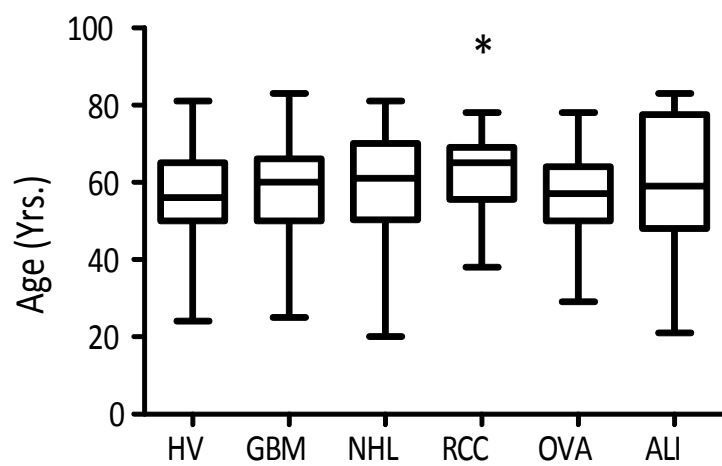

Supplement: Additional file 2: Figure S1 — Age of healthy volunteers and patients. Box and whisker plots show the mean, 25th and 75th percentile, and the range of ages for each cohort. HV- healthy volunteers; GBM- glioblastoma multiforme; NHL-non Hodgkin’s lymphoma; RCC-renal cell carcinoma; OVA-ovarian cancer; ALI- acute lung injury. Asterisk indicates = p < 0.05 vs. HV. [file 2051-1426-1-7-S2.pdf]

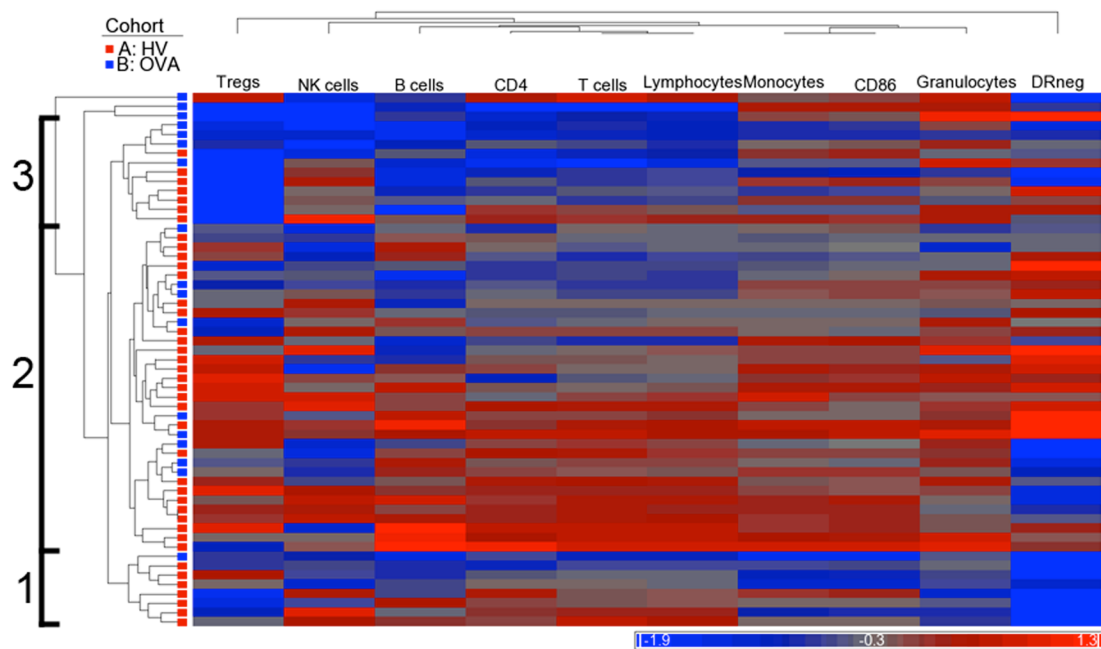

Supplement: Additional file 4: Figure S2 — Hierarchical clustering of ovarian cancer patients. OVA patients were subject to profiling analysis as in Figure 1. Identification of major clusters is indicated at left. A row represents one subject and a column represents one of ten markers measured. The horizontal bar below each plot indicates immune markers decreased (blue) or increased (red) over the mean of the healthy volunteer cohort. (n = 17 OVA and n = 40 HV). [file 2051-1426-1-7-S4.pdf]

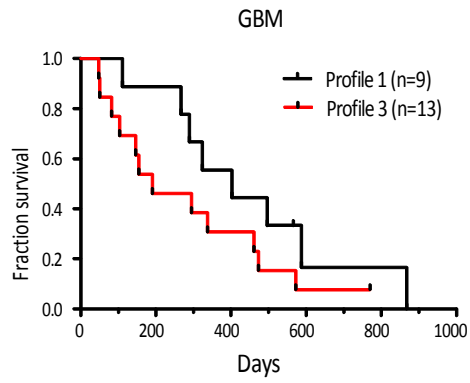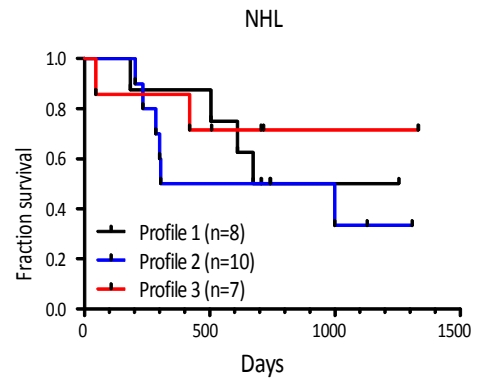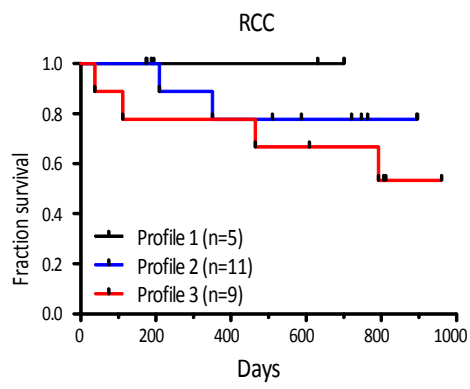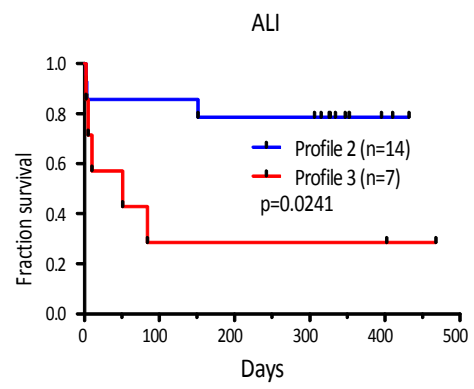

Supplement: Additional file 5: Figure S3 — Survival of patients categorized by immune profile. GBM, NHL, RCC, or ALI patients were categorized to a profile in Figure 1. For each disease, cohorts of patients sharing a profile were plotted for their survival. Note: only profiles with more than three individuals were plotted. [file 2051-1426-1-7-S5.pdf]

Gustafson, et al. Supplemental Figure 1

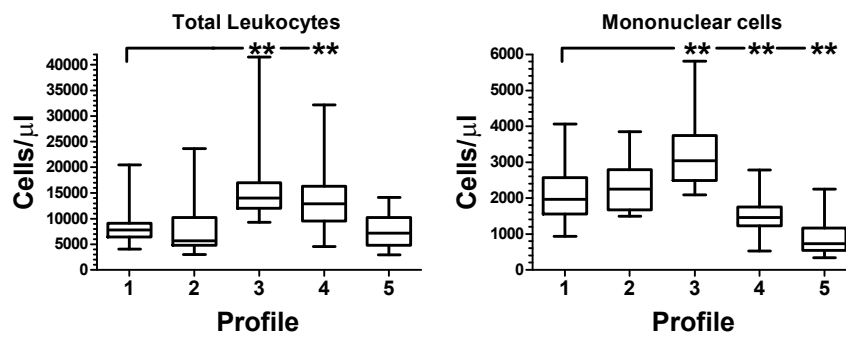

Supplement: Additional file 8: Figure S4 — Immune profile dependent differences in the number of leukocytes and mononuclear cells per μL of blood. Numerical representation of pie charts represented in Figure 3C. Box and whisker plots show the mean, 25th and 75th percentile, and the range of cell counts for each cohort. Differences (p < 0.0001) compared to profile 1 are indicated by ** above the profile. [file 2051-1426-1-7-S8.pdf]

Healthy volunteer

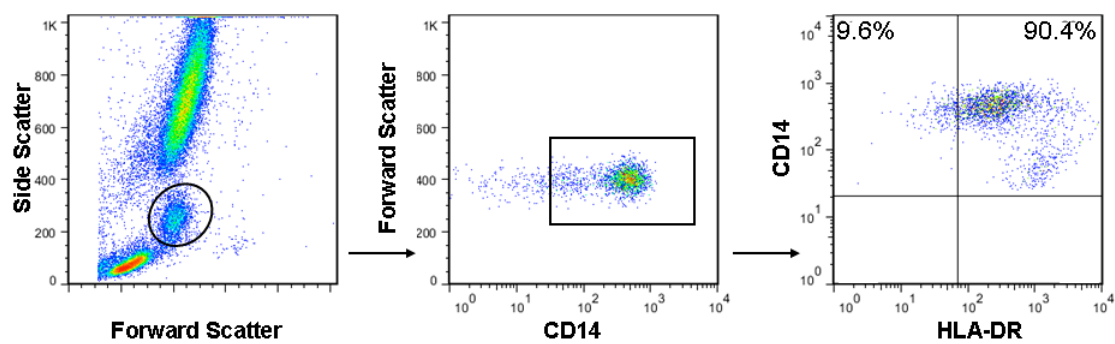

Patient

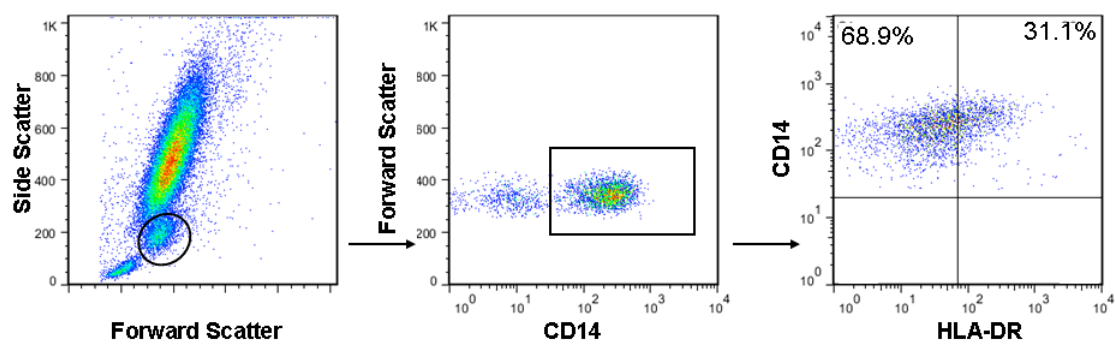

Supplement: Additional file 9: Figure S5 — Gating strategy for CD14+HLA-DRlo/neg monocytes. After preparing the samples for CD14 and HLA-DR whole blood flow cytometry, a gate was placed on the intermediate side scatter and forward scatter cell population. A second gate on cells with low forward scatter and CD14+ was placed. A bivariate plot of CD14 vs. HLA-DR was created. The fraction of the cells in the HLA-DRlo/neg is recorded. A representative plot from a normal healthy volunteer and patient are shown. [file 2051-1426-1-7-S9.pdf]
