# Supplementary material for: Immune monitoring using the predictive power of immune profiles
Source: J Immunother Cancer. 2013 Jun 27;1:7. doi: 10.1186/2051-1426-1-7 (PMC4266565; doi:10.1186/2051-1426-1-7)
Supplement: Additional file 3: Table S2 — Antibodies and reagents used for flow cytometry. [file 2051-1426-1-7-S3.doc]

Table S2. Antibodies and reagents used for flow cytometry

| Reagent | Company | Catalog number |
| --- | --- | --- |
| FACS™ Lysing Solution | BD | 349202 |
| Trucount™ tubes | BD | 340334 |
| BD Multitest CD3/CD16+/CD56/CD45/CD19 | BD | 340500 |
| CD127 PE | BD | 557938 |
| CD4 PerCP | BD | 340671 |
| CD25 APC | BD | 555434 |
| CD3 FITC | BD | 349201 |
| CD3 PE | BD | 340662 |
| CD3 PerCP | BD | 340663 |
| CD3 APC | BD | 340440 |
| CD4 FITC | BD | 555346 |
| CD8 PE | BD | 340046 |
| CD28 APC | BD | 559770 |
| CD152 (CTLA4) PE | BD | 555853 |
| CCR7 FITC | R&D Systems | FAB197F |
| CD62L APC | BD | 559772 |
| CD45RO | BD | 347967 |
| CD8 PerCp | BD | 347314 |
| IGG FITC | BD | 349041 |
| IGG PE | BD | 340761 |
| IGG PERCP | BD | 349044 |
| IGG APC | BD | 340754 |
| CD14 APC | BD | 555399 |
| HLA-DR PerCP | BD | 347364 |
| CD80 FITC | BD | 555683 |
| CD86 PE | BD | 555658 |
| Lineage FITC | BD | 340546 |
| CD33 APC | BD | 551378 |
| CD16 PE | eBioscience | 12-0168-73 |
