# Supplementary material for: Immune monitoring using the predictive power of immune profiles
Source: J Immunother Cancer. 2013 Jun 27;1:7. doi: 10.1186/2051-1426-1-7 (PMC4266565; doi:10.1186/2051-1426-1-7)
Supplement: Additional file 6: Table S3 — P values of the differences in phenotype expression between each immune profile. [file 2051-1426-1-7-S6.doc]

Table S3. P values of the differences in phenotype expression between each immune profile

| **Granulocytes** | **1** | **2** | **3** | **4** | **5** |
| --- | --- | --- | --- | --- | --- |
| HV | 0.0016 | ns | <0.0001 | <0.0001 | 0.0491 |
| 1 |  | 0.0414 | <0.0001 | <0.0001 | ns |
| 2 |  |  | 0.0009 | 0.0002 | ns |
| 3 |  |  |  | ns | 0.0013 |
| 4 |  |  |  |  | 0.0015 |
| **Lymphocytes** | **1** | **2** | **3** | **4** | **5** |
| HV | ns | ns | 0.0110 | <0.0001 | <0.0001 |
| 1 |  | 0.404 | 0.0011 | <0.0001 | <0.0001 |
| 2 |  |  | ns | <0.0001 | <0.0001 |
| 3 |  |  |  | <0.0001 | <0.0001 |
| 4 |  |  |  |  | 0.0371 |
| **Monocytes** | **1** | **2** | **3** | **4** | **5** |
| HV | ns | 0.0051 | <0.0001 | 0.0004 | <0.0001 |
| 1 |  | 0.0006 | <0.0001 | 0.0010 | <0.0001 |
| 2 |  |  | <0.0001 | 0.0001 | 0.0117 |
| 3 |  |  |  | 0.0071 | <0.0001 |
| 4 |  |  |  |  | <0.0001 |
| **T cells** | **1** | **2** | **3** | **4** | **5** |
| HV | ns | ns | 0.0086 | <0.0001 | <0.0001 |
| 1 |  | ns | 0.0018 | <0.0001 | <0.0001 |
| 2 |  |  | ns | <0.0001 | <0.0001 |
| 3 |  |  |  | <0.0001 | <0.0001 |
| 4 |  |  |  |  | 0.0359 |
| **B cells** | **1** | **2** | **3** | **4** | **5** |
| HV | ns | ns | ns | <0.0001 | <0.0001 |
| 1 |  | ns | 0.0121 | <0.0001 | <0.0001 |
| 2 |  |  | 0.0404 | 0.0014 | <0.0001 |
| 3 |  |  |  | 0.0001 | 0.0003 |
| 4 |  |  |  |  | ns |
| **NK cells** | **1** | **2** | **3** | **4** | **5** |
| HV | ns | ns | ns | <0.0001 | 0.0002 |
| 1 |  | ns | ns | <0.0001 | 0.0002 |
| 2 |  |  | ns | 0.0002 | 0.0011 |
| 3 |  |  |  | 0.0012 | 0.0072 |
| 4 |  |  |  |  | ns |
| **CD4+ T cells** | **1** | **2** | **3** | **4** | **5** |
| HV | ns | ns | 0.0016 | <0.0001 | <0.0001 |
| 1 |  | ns | 0.0016 | <0.0001 | <0.0001 |
| 2 |  |  | ns | <0.0001 | 0.0001 |
| 3 |  |  |  | <0.0001 | <0.0001 |
| 4 |  |  |  |  | ns |
| **Regulatory T cells** | **1** | **2** | **3** | **4** | **5** |
| HV | ns | ns | 0.0010 | <0.0001 | 0.0332 |
| 1 |  | ns | 0.0016 | <0.0001 | 0.0074 |
| 2 |  |  | 0.0156 | 0.0006 | 0.0298 |
| 3 |  |  |  | <0.0001 | 0.0005 |
| 4 |  |  |  |  | ns |
| **CD14^+^HLA-DR^lo/neg^ monocytes** | **1** | **2** | **3** | **4** | **5** |
| HV | 0.0017 | <0.0001 | <0.0001 | <0.0001 | ns |
| 1 |  | <0.0001 | <0.0001 | <0.0001 | ns |
| 2 |  |  | <0.0001 | <0.0001 | 0.0001 |
| 3 |  |  |  | ns | <0.0001 |
| 4 |  |  |  |  | <0.0001 |
| **CD86+ monocytes** | **1** | **2** | **3** | **4** | **5** |
| HV | ns | 0.0060 | <0.0001 | ns | <0.0001 |
| 1 |  | 0.0010 | <0.0001 | ns | <0.0001 |
| 2 |  |  | <0.0001 | 0.0080 | 0.0061 |
| 3 |  |  |  | 0.0019 | <0.0001 |
| 4 |  |  |  |  | <0.0001 |

ns= p>0.05. NOTE: P values obtained using T test of unpaired samples with
