# Supplementary material for: Immune monitoring using the predictive power of immune profiles
Source: J Immunother Cancer. 2013 Jun 27;1:7. doi: 10.1186/2051-1426-1-7 (PMC4266565; doi:10.1186/2051-1426-1-7)
Supplement: Additional file 7: Table S4 — Frequency of phenotypes for each pathology in this study. [file 2051-1426-1-7-S7.doc]

Table S4. Frequency of phenotypes for each pathology in this study

|  | HV | GBM | NHL | RCC | OVA | ALI |
| --- | --- | --- | --- | --- | --- | --- |
| Granulocytes (FSC vs SSC)  Mean (% of WBCs)  St. Dev.  Range | **65.0**  6.8  48.7-79.3 | **75.7**  12.1  38.9-89.3 | **85.6**  3.4  80.8-95.0 | **70.7**  10.0  35.9-94.9 | **73.8**  6.9  61.6-87.4 | **82.3**  8.4  63.6-93.2 |
| Lymphocytes (FSC vs SSC)  Mean (% of WBCs)  St. Dev.  Range | **25.0**  6.3  13.7-40.5 | **14.2**  10.8  1.6-  45.8 | **10.3**  3.7  4.0-  15.9 | **19.5**  9.2  2.6-  54.3 | **18.2**  6.2  7.2-  28.5 | **10.1**  7.0  1.9-  26.2 |
| Monocytes (FSC vs SSC)  Mean (% of WBCs)  St. Dev.  Range | **7.2**  1.8  4.5-12.4 | **6.2**  2.4  2.8-14.6 | **4.1**  1.6  0.9-7.6 | **7.8**  2.0  2.2-10.8 | **6.1**  1.8  3.5-9.0 | **5.3**  2.6  0.9-10.3 |
| T cells (CD3^+^)  Mean (Cells/μ)  St. Dev.  Range | **1258**  387  693-2182 | **939**  635  96-  2567 | **778**  580  161-2497 | **1334**  504  533-2319 | **1026**  468  317-2045 | **834**  673  127-3023 |
| B cells (CD19^+^)  Mean (Cells/μl)  St. Dev.  Range | **243**  127  62-572 | **226**  172  25-793 | **155**  182  7-648 | **205**  210  47-1057 | **189**  92  87-373 | **345**  843  15-4208 |
| NK cells (CD56^+^CD16^+^)  Mean (Cells/μl)  St. Dev.  Range | **222**  102  80-459 | **179**  203  8-1059 | **222**  141  34-781 | **184**  84  66-384 | **123**  59  39-234 | **128**  110  14-482 |
| CD4^+^ T cells  Mean (Cells/μl)  St. Dev.  Range | **932**  314  358-2098 | **626**  446  81-  1764 | **506**  431  92-  2084 | **940**  341  431-1725 | **729**  303  303-1408 | **601**  510  84-  2079 |
| Tregs (CD4^+^CD25^+^CD127^lo^)  Mean (% of CD4^+^ T cells)  St. Dev.  Range | **3.3**  2.2  0.6-11.8 | **6.6**  3.8  0.7-14.4 | **5.7**  3.7  0.9-14.1 | **4.8**  2.9  0.4-11.4 | **2.8**  0.9  0.7-4.1 | **4.0**  2.8  0.4-11.2 |
| CD14^+^HLA-DR^lo/neg^  Mean (% CD14^+^ monocytes)  St. Dev.  Range | **10.7**  8.4  0.7-  40.4 | **31.2**  23.9  0.8-  94.0 | **23.0**  17.8  2.6-  69.1 | **35.5**  24.9  5.3-  93.9 | **11.9**  10.9  0.3-  43.6 | **67.1**  13.1  30.9-83.1 |

HV= Healthy volunteers; GBM = glioblastoma; NHL = non-Hodgkin’s lymphoma; RCC = renal cell carcinoma; OVA = ovarian carcinoma; ALI = acute lung injury
